# Leadless Pacemaker Implantation During Extraction in Patients with Active Infection: A Comprehensive Analysis of Safety, Patient Benefits and Costs

**DOI:** 10.3390/jcm14155450

**Published:** 2025-08-02

**Authors:** Aviv Solomon, Maor Tzuberi, Anat Berkovitch, Eran Hoch, Roy Beinart, Eyal Nof

**Affiliations:** 1 The Chaim Sheba Medical Center, Leviev Heart Institute, Tel Hashomer, Ramat Gan 5262000, Israeleyal.nof@sheba.health.gov.il (E.N.); 2 School of Medicine, Tel Aviv University, Tel Aviv 69978, Israel

**Keywords:** leadless pacemaker, extraction, cardiac implantable electronic device, infection

## Abstract

**Background:** Cardiac implantable electronic device (CIED) infections necessitate extraction and subsequent pacing interventions. Conventional methods after removing the infected CIED system involve temporary or semi-permanent pacing followed by delayed permanent pacemaker (PPM) implantation. Leadless pacemakers (LPs) may offer an alternative, allowing immediate PPM implantation without increasing infection risks. Our objective is to evaluate the safety and cost-effectiveness of LP implantation during the same procedure of CIED extraction, compared to conventional two-stage approaches. **Methods:** Pacemaker-dependent patients with systemic or pocket infection undergoing device extraction and LP implantation during the same procedure at Sheba Medical Center, Israel, were compared to a historical group of patients undergoing a semi-permanent (SP) pacemaker implantation during the procedure, followed by a permanent pacemaker implantation. **Results:** The cohort included 87 patients, 45 undergoing LP implantation and 42 SP implantation during the extraction procedure. The LP group demonstrated shorter intensive care unit stay (1 ± 3 days vs. 7 ± 12 days, *p* < 0.001) and overall hospital days (11 ± 24 days vs. 17 ± 17 days, *p* < 0.001). Rates of infection relapse and one-year mortality were comparable between groups. Economic analysis revealed comparable total costs, despite the higher initial expense of LPs. **Conclusions:** LP implantation during CIED extraction offers significant clinical and logistical advantages, including reduced hospital stays and streamlined treatment, with comparable safety and cost-effectiveness to conventional approaches.

## 1. Introduction

Cardiac implantable electronic device (CIED) dependent patients present a unique problem in the setting of CIED-related infection or persistent bacteremia requiring extraction. These patients require temporary device implantation prolonging hospitalization, elevating the risk for relapse infection and further complications. Typically, these patients receive a semi-permanent (SP) device (i.e., a transvenous “screw in” lead) via the right internal jugular vein (RIJV) connected to the pulse generator (PG) which is placed above the skin. In this setting, current practice standards support waiting for a minimum of 72 h for negative blood cultures and up to 4 weeks of antibiotic therapy before reimplanting a permanent pacemaker (PPM) [1]. This “two-stage” procedure by itself prolongs the hospitalization course. Furthermore, in many centers, pacemaker-dependent patients with an SP device need to be admitted to an intensive care unit (ICU) or at minimum to a monitored bed. This imposes a significant burden and cost on the health system.

Leadless pacing (LP) systems are implanted directly into the right ventricle (RV) using a catheter delivery system [2,3]. LP eliminates the risk of pocket infection and lead-associated endocarditis and serves as a permanent pacing device [4,5]. Thus, this device may serve as a solution for patients presenting with CIED infection. However, LPs are more expensive than transvenous pacemakers. To date, several studies have reported safety results comparing the two approaches [1,4,6,7,8], but there is no study that has compared the costs.

The aim of this study is to evaluate the safety and cost-effectiveness of LP implantation immediately post-extraction in the setting of an acute CIED infection.

## 2. Methods

Pacemaker-dependent patients with systemic or pocket infection undergoing transvenous lead extraction (TLE) and LP implantation during the same procedure at Sheba Medical Center, Israel, were compared to a matched historical group of patients undergoing an SP pacemaker during the procedure. This study was approved by the Institutional Helsinki review board of our hospital.

All patients included in this study were indicated for a pacemaker and not contra-indicated for an LP. LP included the Micra transcatheter pacing system (Medtronic Inc, Minneapolis, MN, USA) or the AVEIR transcatheter pacing system (Abbott Laboratories, Abbott Park, IL, USA).

### 2.1. Extraction Procedure

All TLE procedures were performed by qualified experienced operators, with a cardiothoracic surgeon immediately available on-site. Patients were under general anesthesia, with hemodynamic monitoring and transesophageal echo-cardiography. A large-bore femoral venous access was inserted in all patients in case femoral bailout was warranted and for the SVC bridging balloon wire, after it became available. A quadripolar catheter was inserted into the right ventricle via the right femoral vein and connected to an external pacemaker. The pacemaker was programmed in an asynchronous pacing mode during the procedure. A stepwise approach for extracting the leads was used in all patients as previously described by our group [9]. The TLE procedure was terminated after complete removal of the leads, when lead fragments could not be removed or in the event of major complication.

Complications were divided into major complications (defined as life-threatening, requiring surgical intervention or resulting in death). Complications that did not meet the major complication criteria were classified as minor complications. Success or failure was defined by the radiological findings and not clinical success results. Patients were divided into three groups depending on the outcome of the extraction procedure: “Complete success” was classified as the removal of the entire lead system (including “lead tips”) being achieved. “Partial success” was defined as when most of the lead was removed, leaving at most 4 cm of coil and/or insulation and/or lead tip, and “Failure” was definite if a ≥ 4 cm tip remained.

### 2.2. LP Implantation Procedure

In cases of LP implantation, the procedure was performed via the right femoral vein at the end of the TLE procedure. The LP was placed in the right ventricle (RV) septum (for MICRA AV) or RV apical septum (for AVEIR VR). MICRA was chosen for patients in sinus rhythm while AVEIR was chosen for patients in permanent atrial fibrillation. The DR AVIER was not available in our institution at the time of this study and therefore was not implanted. After the LP was deployed and sensing and pacing amplitude were found to be acceptable, the quadripolar catheter which served as the temporary pacing wire was removed. The groin sheaths were taken out and a “figure of 8” suture was placed.

### 2.3. SP Implantation Procedure

In case an SP was implanted, an active fixation lead was placed into the RV via the RIJV. The lead was then connected to a PG fixed with a suture to the skin. Once infectious disease clearance was given (minimum of post-extraction 72 h of negative blood cultures), a transvenous permanent pacemaker was implanted on the contralateral side and the SP was removed.

### 2.4. Study Endpoints

The primary efficacy endpoint included days in ICU or monitored bed and time to discharge. The primary safety endpoint included relapse of infection in the first three months. The secondary safety endpoint included one year mortality.

In a secondary explorative analysis, the clinical outcomes of the LP-enrolled study patients were compared to matched patients undergoing SP implantation during TLE (leading later to a conventional pacemaker after infectious control was achieved). This analysis also included the cost-effectiveness of both approaches. The comparison of the cost-effectiveness included costs of the LP vs. SP+ permanent pacemaker implantation, the cost of the procedure and cost of days in the ICU and in the non-ICU department. The Sheba Medical Center’s financial department assisted in estimating the expenditures.

A sub-analysis excluding patients with CRT/D (cardiac resynchronization therapy) devices was performed as these, due to the nature of their disease, were less likely to receive a leadless pacemaker.

### 2.5. Statistical Analysis

Categorical variables were expressed as frequency and percentages. A chi-square test was used to evaluate the association between these variables. The distribution of continuous variables was assessed using Kolmogorov–Smirnov test. Normally distributed continuous variables were described as mean and standard deviations (SDs), these variables were compared using the independent samples *t*-test. Non-normally distributed continuous variables were expressed as median and interquartile ranges (IQR); the Mann–Whitney U test was used for comparison. Survival curves were plotted using the Kaplan–Meier method. A two-tailed *p*-value of <0.05 was considered significant for all analyses. All analyses were performed with IBM SPSS 29.0 software (SPSS Inc., Chicago, IL, USA).

### 2.6. Cost Analysis

To ensure accurate financial comparisons, we utilized detailed cost breakdowns from Sheba Medical Center’s financial systems. These estimates include direct costs (LP and temporary pacemaker, implantation, Internal ward and ICU hospitalization). Procedure related costs were mapped to diagnosis-related group codes aligned with local standards. We acknowledge that these costs may vary across international settings. However, they provide an estimate of economic impact within a tertiary-care center.

## 3. Results

### 3.1. Baseline Patient Characteristics

From September 2019 to December 2023, 45 patients received LPs in the extraction procedure (5 AVEIR and 40 Micra). These patients were compared to the SP group, consisting of 42 patients who received a temporary transvenous pacemaker during the extraction procedure, followed by a PPM implantation. Baseline characteristics as well as the type of infection and indication for extraction are presented in Table 1. The median follow-up time was 488 days in the LP and 2302 days in the SP group.

Our cohort’s mean age was 74 ± 13 and predominantly male (78%). Cardiac risk factors were relatively common and included hypertension (69%), diabetes mellitus (45%) and chronic kidney disease (26%); however, no significant difference was observed between the groups (Table 1). Left ventricular ejection fraction (LVEF) was significantly higher in the LP group (52 ± 9 vs. 45 ± 13%, *p* = 0.018). There were more patients with CRTD in the SP group (Table 1).

All patients who presented with CIED infection were classified as either systemic (26%), pocket (38%) or combined (33%). At the time of extraction, 24 (28%) of the patients had documented vegetation on their leads on transesophageal echocardiography (TEE). The predominant bacteremia in both groups was Staphylococcus (54%). The prevailing pacing indications were AV block (81.59%) or sick sinus syndrome (8%).

### 3.2. Extraction Procedure

The procedural techniques used are presented in Table 2. Simple traction was used in 10 (24%) vs. 18 (40%) patients of the SP group and the LP group, respectively (*p* = 0.106). The use of laser sheath was higher in the SP group (57 vs. 7%, *p* < 0.001), while there was more use of Sub C TightRail in the LP group (0 vs. 44%, *p* < 0.001).

The extraction procedure was completely successful in the majority of patients (92%), with partial success in the remaining 7 patients (8%). There were no major procedural complications in both groups of this cohort or need for re-intervention.

### 3.3. Pacemaker Implantation

All LPs and SPs were successfully implanted. There were no major procedural complications or dislodgments of the SPs or LPs.

### 3.4. Primary and Secondary Endpoints

**Figure 1:**
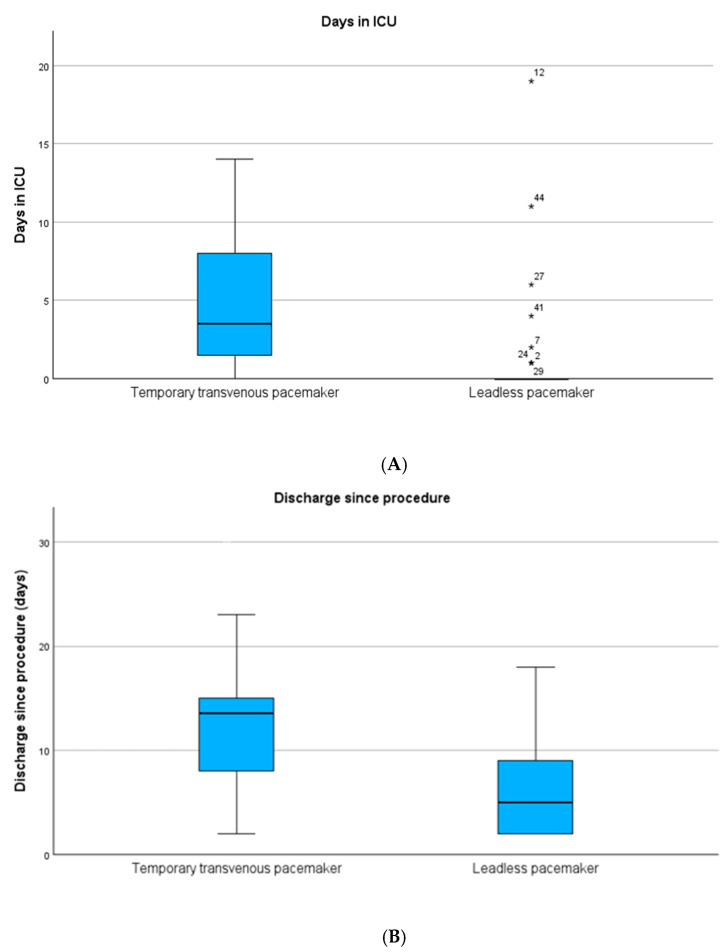
Primary outcome. (**A**). Comparison of days to discharge after the index procedure between the SP and LP groups and (**B**). comparison of days in ICU between the SP and LP groups, showing higher stay in the SP group. * Patients beyond the IQR range.

**Figure 2:**
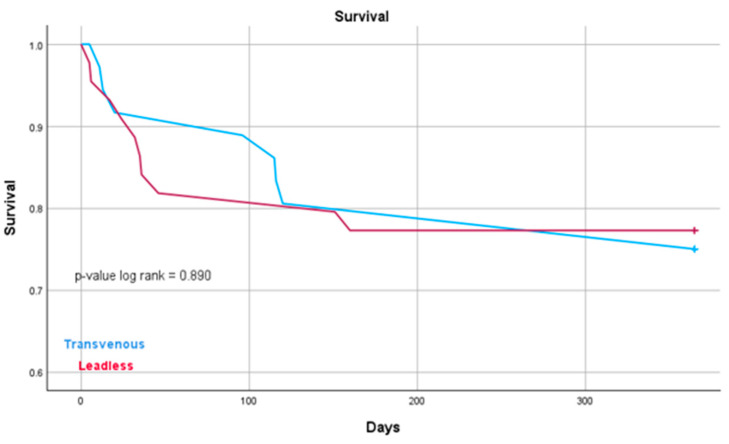
Overall survival. Kaplan–Meier curves show no statistically significant difference in overall survival between the SP and LP groups. OS = overall survival.

A significantly longer ICU stay was observed in the SP pacemaker group compared to the LP group (7 ± 12 vs. 1 ± 3 days, *p* < 0.001), as well as a longer total hospital stay after the extraction procedure (17 ± 17 vs. 11 ± 24 days, *p* < 0.001) (Table 3, Figure 1). A sub-analysis excluding patients with CRT/CRTD prior to the extraction procedure yielded similar results, maintaining statistical significance for all outcomes except for ICU days (0.58 vs. 8.06, *p* = 0.099). The overall one-year survival was similar between the groups (24 vs. 23%, *p* = 0.994) (Table 3, Figure 2). In the SP group, the mean time for PPM implantation was 8 ± 10 days following the extraction procedure.

Three patients, two in the LP group and one in the SP pacemaker group, had re-infection. Two had the same bacteremia as before the extraction procedure (2 months and 5 years later, respectively), and upon reviewing their medical records, it appears that osteomyelitis, rather than CIED infection, was the cause of both re-infections. The third patient had lead extraction failure. One patient in the LP group died 151 days after the extraction procedure while in hospital, due to a different bacteria from the original CIED infection and was therefore excluded from the analysis. He died due to renal failure.

### 3.5. Cost Analysis of Both Approaches

**Figure 3:**
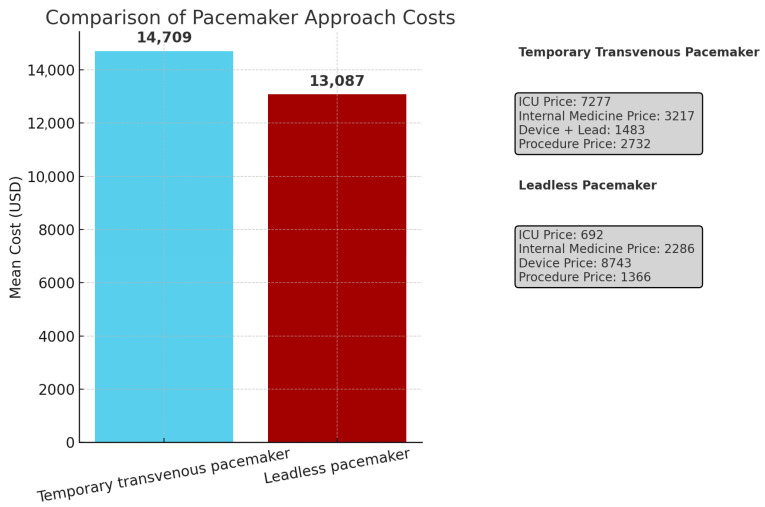
Cost analysis. Comparison of total cost and cost components between temporary transvenous pacemaker and leadless pacemaker approaches. The mean cost of ICU hospitalization in the LP group was significantly lower than the cost in the SP group. The mean total costs were not significantly different between the groups.

A comparison of the costs of both approaches is shown in Table 4 and in Figure 3. As expected, the mean cost of ICU hospitalization in the LP group was significantly lower than the cost in the SP group (USD 692 vs. USD 7277, *p* < 0.001). There was a smaller significant difference in the LP and SP groups regarding the cost of internal medicine hospitalization days (USD 3217 vs. USD 2286, *p* = 0.031). The mean total costs were not significantly different between the groups.

## 4. Discussion

The main finding of our study is that LP implantation during extraction for CIED infection reduces the burden on health systems compared to SP implantation followed by PPM implantation. Although LPs are significantly more expensive than traditional transvenous pacemakers, implantation of LPs in this specific setting shortened ICU and total hospitalization stay, resulting in overall similar costs.

Our study also shows that this approach is safe. LPs have been demonstrated to be a safe approach for the treatment of pacemaker-dependent patients with an active infection requiring extraction. In a study of 86 patients who underwent LP implantation in the same procedure as transvenous lead extraction, no recurrent infections were found throughout the median follow-up period of 163 days [6]. Chang et al. reported on 17 patients who received an LP during infected CIED extraction, none of them had a recurrent infection [1]. These and other observational data [4,7] confirm that LP systems are much less prone to infection and therefore could be a valuable solution for patients undergoing extraction. A reduction in ICU stay has also been demonstrated in a multicenter prospective study of 45 pacemaker-dependent patients with device infection [8]. Patients who underwent LP implantation immediately after extraction had a significantly shorter ICU stay compared to a historical cohort of patients who had postponed reimplantation.

Our study adds to this body of literature by showing that the costs of the two approaches were the same, while the LP approach saved precious staff time and freed up hospital beds (including in ICU, where resources are particularly scarce). These findings are important when a physician needs to decide between the two approaches. Implanting an LP is significantly more expensive than a traditional transvenous device, and it was not clear if the costs would be offset. While the costs of hospital beds and the cost of LPs may differ from country to country, our results can be applied to any country as the rate of LP cost to transvenous pacing cost is more or less similar, as are costs of monitored bed vs. regular beds and of course reduction in hospital stay. Reducing hospitalization also provides important clinical benefits such as minimizing patient exposure to hospital-associated risks, including secondary infections (even more in ICU wards), and facilitates earlier return to daily activities. LP may also have the advantage of reducing tricuspid regurgitation in the long term follow up [10].

A two-stage procedure approach also exposes patients to additional procedural risks. Moreover, it necessitates temporary pacing devices for a prolonged time in patients, limiting their mobility and thus imposing substantial inconvenience. As temporary pacing devices are inherently less stable, they are more prone to complications.

In all our cases, patients were implanted with a temporary pacing system during the extraction procedure and not immediately with an LP. Our routine choice for temporary pacing is a quadripolar catheter connected to an external pacemaker. Our experience shows that these catheters are more stable and less likely to dislodge during the extraction procedure than a balloon-tipped temporary wire pacemaker. The reason for doing so and not immediately implanting an LP is to prevent potential LP dislodgment during the extraction procedure which at times can result in RV invagination by pulling the lead back during advancement of the extraction tool/sheath. This happened in the case series mentioned above by Chang et al [1]. Moreover, in case a major complication occurs, and sternotomy is necessary, we implant an epicardial system instead of an LP. In such a scenario, there is eventually no need for an LP, and therefore, implanting one ahead of the procedure might turn out to be unnecessary and a waste of resources. Fortunately, none of our patients included in this study ended up needing urgent sternotomy.

LPs have been shown to have a low risk of device infection [11,12,13]. The lower risk of infection is likely related to smaller device size, no pocket, no lead, parylene coating, turbulent environment and reduced handling [14]. In particular, the device interface with the blood system is significantly lower with an LP than with a full transvenous system with leads. Furthermore, over time the LP has been shown to be encapsulated with tissue which most probably prevents bacteria colonization [1]. Another source of re-infection is the pacemaker pocket which of course is absent in LP cases.

In our study, there was more use of laser sheath in the SP group and more use of TightRail (specifically SubC) in the LP group. This merely reflects our current shift and preference over the years of our first choice extraction tool but did not have any effect on the extraction success results.

There were more patients with CRTD in the SP group. We did include patients with CRTD in the initial analysis as some patients were implanted with leadless pacemakers in spite of undergoing CRTD extraction due to multiple reasons (mainly clinical re-assessment of need for CRTD).

### 4.1. Study Limitations

While this study provides evidence supporting the LP-first strategy, limitations include its retrospective nature, single-center design and relatively small sample size. Patients with CRTD were less likely to receive an LP as most were planned for reimplantation of a CRTD post-extraction. Owing to the matching, differences on observed characteristics are balanced; however, patients could differ on unobserved characteristics.

Of note, follow-up times differed between groups as patients with SPs had a much longer follow up. However, even if censored for same follow-up durations, the results do not differ.

Long-term cost-effectiveness analyses and larger multicenter studies are warranted to validate these findings further.

Additionally, reliance on historical controls for the SP group may introduce confounding variables due to evolving clinical practices. The lack of formal quality of life assessments or patient-reported outcomes limits our ability to evaluate the patients’ restoration of mobility and independence.

### 4.2. Future Directions

We recommend a multicenter randomized controlled trial comparing LPs with conventional pacing systems in terms of patient outcomes, cost-analysis and hospital resource utilization. In addition, incorporating functional assessments and quality-of-life metrics will enhance understanding of the patient-centered impact of LP technology.

## 5. Conclusions

In summary, adopting LPs during CIED extraction represents a paradigm shift in managing device-related infections. The ability to provide definitive therapy during the index procedure could not only enhance patient care but also optimize resource utilization. This approach aligns with contemporary healthcare priorities of delivering cost-efficient, high-quality care.

## Figures and Tables

**Table 1:** Baseline characteristics.

	Entire Cohort (n = 87)	Temporary Transvenous Pacemaker (n = 42)	Leadless Pacemaker (n = 45)	*p*-Value
**Demographics**				
Age, years, mean (SD)	74 (13)	72 (14)	76 (12)	0.073
Female gender, n (%)	19 (21.8)	8 (19.0)	11 (24.4)	0.543
Diabetes mellitus, n (%)	39 (45.3)	21 (50)	18 (40.9)	0.397
Hypertension, n (%)	59 (68.6)	29 (69)	30 (68.2)	0.931
CVA/TIA, n (%)	23 (26.7)	12 (28.6)	11 (25.0)	0.283
Chronic atrial fibrillation, n (%)	36 (41.4)	20 (47.6)	16 (35.6)	0.254
Chronic kidney disease, n (%)	22 (25.6)	9 (21.4)	13 (29.5)	0.388
LVEF, mean (SD)	49 (12)	45 (13)	52 (9)	**0.018**
**Cardiomyopathy, n (%)**				**0.015**
Ischemic	11 (13.8)	6 (16.7)	5 (11.4)	
Dilated	4 (5.0)	4 (11.1)	0 (0.0)	
Hypertrophic	3 (3.8)	3 (8.3)	0 (0.0)	
COPD, n (%)	13 (15.3)	7 (17.1)	6 (13.6)	0.660
Severe pulmonary hypertension, n (%)	15 (19.2)	7 (20.6)	8 (18.2)	0.789
Valve replacement, n (%)	25 (40.3)	9 (21.4)	16 (35.6)	0.396
Number of electrodes (Median)	2.3 (2)	2.5 (2.5)	2.1 (2)	**0.011**
**Device type, n (%)**				**0.013**
CRTD	18 (20.9)	14 (33.3)	4 (9.1)	
CRTP	10 (11.6)	5 (11.9)	5 (11.4)	
Single chamber	10 (11.6)	7 (16.7)	3 (6.8)	
Dual chamber	47 (54.7)	16 (38.1)	31 (70.4)	
**Pacing indication, n (%)**				0.453
SSS	7 (8.04)	3 (7.14)	4 (8.9)	
High degree AV block	53 (60.91)	29 (69.04)	24 (53.3)	
Atrial fibrillation with AV block	18 (20.68)	6 (14.28)	12 (26.7)	
**Indication for extraction, n (%)**				0.139
Pocket infection	33 (37.9)	19 (45.2)	14 (31.1)	
Systemic infection	23 (26.4)	8 (19.0)	15 (33.3)	
Combined pocket + systemic	28 (32.2)	15 (35.7)	13 (28.9)	
**Type of infection, n (%)**				0.113
Streptococcus	4 (5.9)	2 (5.1)	2 (4.6)	
MSSA	10 (14.7)	7 (17.9)	3 (6.8)	
Staphylococcus (other)	29 (42.6)	12 (31.2)	17 (39.1)	
Enterococcus	10 (14.7)	2 (5.1)	8 (18.2)	

COPD = chronic obstructive pulmonary disease; CVA = cerebrovascular accident; CRTD = cardiac resynchronization therapy defibrillator; CRTP = cardiac resynchronization therapy pacemaker; LVEF = left ventricular ejection fraction; MSSA = methicillin-sensitive Staphylococcus aureus; n = number; SD = standard deviation; SSS = sick sinus syndrome; TIA = transient ischemic attack.

**Table 2:** Extraction procedure.

	Temporary Transvenous Pacemaker Patients (n = 42)	Leadless Pacemaker Patients (n = 45)	*p*-Value
Simple Traction (%)	10 (23.8)	18 (40)	0.106
Laser (%)	24 (57.1)	3 (6.7)	<0.001
TightRail (%)	11 (26.2)	9 (20)	0.493
Sub C TightRail (%)	0 (0)	20 (44.4)	<0.001
Femoral Work Station (%)	3 (7.1)	1 (2.2)	0.273

**Table 3:** Primary outcome.

	Temporary Transvenous Pacemaker Patients (n = 32)	Leadless Pacemaker Patients (n = 42)	*p*-Value
Days in ICU, mean (SD)	7 (12)	1 (3)	**<0.001**
Days in hospital, mean (SD)	17 (17)	11 (24)	**<0.001**
1-year mortality, n (%)	9 (23.7)	10 (22.7)	0.890

n = number; SD = standard deviation; ICU = intensive care unit.

**Table 4:** Costs.

	Temporary Transvenous Pacemaker Approach (USD) (n = 32)	Leadless Pacemaker Approach (USD) (n = 41)	*p*-Value
ICU, mean (SD)	7277 (13,457)	692 (2210)	**<0.001**
Internal Department, mean (SD)	3217 (2789)	2286 (3378)	**0.031**
Device + Temporary Screw in Lead	1483	8743	
Procedure	2732	1366	
Total, mean	14,709	13,087	0.321

ILS to USD conversion 3.66:1 ratio (30 November 2024).

## Data Availability

No new data were created.
